# A Rare Variation of Coeliac Trunk Hexafurcation

**DOI:** 10.3390/diagnostics16050701

**Published:** 2026-02-27

**Authors:** George Triantafyllou, Nikolaos-Achilleas Arkoudis, Georgios Velonakis, Alexandros Samolis, Orestis Lyros, Panagiotis Kokoropoulos, Maria Piagkou

**Affiliations:** 1 Department of Anatomy, School of Medicine, Faculty of Health Sciences, National and Kapodistrian University of Athens, 11527 Athens, Greece; georgerose406@gmail.com (G.T.); alexsamolis@me.com (A.S.); 2 Research Unit of Radiology and Medical Imaging, National and Kapodistrian University of Athens, 11527 Athens, Greece; nick.arkoudis@gmail.com (N.-A.A.); gvelonakis@med.uoa.gr (G.V.); 3 Second Department of Radiology, General University Hospital “Attikon”, National and Kapodistrian University of Athens, 12462 Athens, Greece; 4 Fourth Department of Surgery, General University Hospital “Attikon”, National and Kapodistrian University of Athens, 12462 Athens, Greece; lyrosrestis@gmail.com (O.L.); kokoropoulos@yahoo.gr (P.K.)

**Keywords:** coeliac trunk, hexafurcation, inferior phrenic artery, left gastric artery, common hepatic artery, splenic artery, pancreatic arteries

## Abstract

We report an incidental finding of a coeliac trunk (CeT) hexafurcation in a 62-year-old female during computed tomography angiography, where the trunk sequentially branches off the left and right inferior phrenic arteries (IPAs) and the left gastric artery (LGA) as collateral branches, before a terminal trifurcation into the common hepatic (CHA), splenic (SA), and dorsal pancreatic (DPA) arteries. While the CeT typically trifurcates, hexafurcation is exceedingly rare. This variation likely stems from the failure of standard regression of primitive splanchnic arteries. The aberrant branches (IPA and DPA) are involved in numerous surgical and interventional procedures. Therefore, this image emphasizes the necessity of high-resolution preoperative imaging and three-dimensional reconstruction to identify complex vascular morphologies

**Figure 1:**
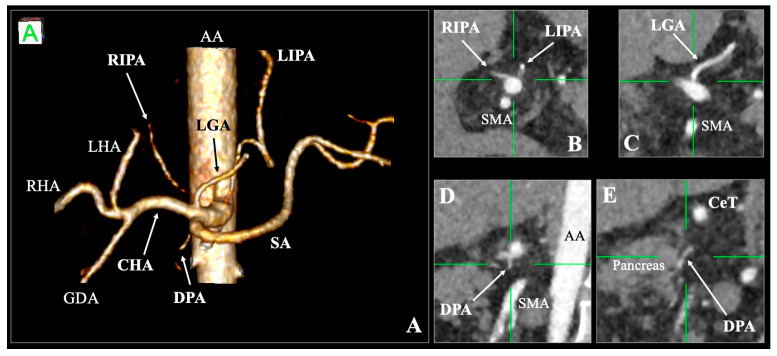
Three-dimensional (**A**) and multiplanar (**B**–**E**) reconstructions of the arterial anatomy of the patient. A hexafurcated coeliac trunk (CeT) can be observed in the three-dimensional volume reconstruction. The right and left inferior phrenic arteries (RIPA and LIPA) are identified in coronal reconstructions (**B**), as well as the left gastric artery (**C**). Lastly, the dorsal pancreatic artery (DPA) origin and course posteriorly to the pancreas can be observed in sagittal reconstructions (**D**,**E**). The image was identified during an abdominal computed tomography angiography assessment of a 62-year-old female patient. The examination was performed using a multidetector CT scanner (*Canon Medical Systems*, *Tochigi*, *Japan*). Imaging was acquired using a contrast-enhanced biphasic abdominal CT protocol. Intravenous iodinated contrast medium (370 mg iodine/mL) was administered via a power injector at a rate of approximately 4 mL/s, followed by a saline flush. Arterial phase acquisition was acquired using bolus tracking, with the region of interest placed in the abdominal aorta (AA). The scan was evaluated using Horos software version 3.3.6 (*Horos Project*, *New York*, *NY*, *USA*). Multiplanar reconstructions (axial, coronal, and sagittal) and three-dimensional volume-rendered images were used to assess the vascular anatomy. Typically, the CeT branches off the left gastric (LGA), the common hepatic (CHA) and the splenic (SA) arteries [1]. Numerous variations on this branching pattern have been described. *Bergman’s Comprehensive Encyclopedia of Human Anatomic Variations* states that the CeT can range from absence (all the arteries arising from the aorta) to six branches (three typical and three additional) [2]. The most common variation is considered to be the tetrafurcation of the CeT, with the additional branch corresponding to either one of the inferior phrenic arteries (IPA), the dorsal pancreatic artery (DPA) or the gastroduodenal artery [2]. During the recent meta-analysis of CeT anatomy, the variations were classified from type 0 (absence of the trunk) to type 4 (three typical and one additional branch). The typical configuration (type 3) had a pooled prevalence of 83.15% [3]. The presence of accessory branches arising from the CeT (type 4) was considered infrequent, with a pooled prevalence of 1.80% [3]—excluding the IPA and DPA, which very commonly arise from the CeT [4,5]. However, during the systematic review, sporadic cases reported the pentafurcation or hexafurcation of the CeT [3]. The current case corresponds to a hexafurcated variant of the CeT with the three typical (LGA, CHA and SA) and three additional branches (LIPA, RIPA and DPA). Manta et al. [6] reported two cases of CeT hexafurcation and reviewed similar reports. They were able to identify eight cases in the current literature. However, only one of them presented photographic evidence of the variation [7]. Therefore, according to Manta et al. [6] and our observations, only four evidence-based cases reporting CeT hexafurcation were previously reported. Nevertheless, a single case of CeT heptafurcation was also recorded recently [8]. The embryological mechanism for this rare hexafurcation is best explained by the longitudinal ventral anastomosis theory. During the embryonic stage, the 10th through 13th metameric ventral splanchnic arteries arise from the aorta to form the LGA, SA, CHA, and superior mesenteric arteries, respectively [3]. These primitive vessels are interconnected by a longitudinal ventral anastomosis located in front of the aorta (the so-called arc of Bühler). In the reported case, the hexafurcation likely resulted from a failure of standard regression, where branches that normally migrate to secondary origins (DPA and IPA) maintained their primary connections directly to the coeliac axis [4,5]. The clinical and surgical significance of identifying a hexafurcated CeT variant is substantial, as precise knowledge of these arterial patterns is critical for preoperative planning and preventing intraoperative complications. The presence of the DPA as a direct terminal branch of the CeT is particularly vital in pancreatic surgery. During a pancreaticoduodenectomy, the DPA must be identified early to facilitate ligation, which reduces blood loss and ensures complete arterial exclusion of the pancreatic head [9,10]. In laparoscopic procedures, a CeT-derived DPA can be easily overlooked, leading to inadvertent and potentially uncontrollable bleeding [9,10]. Furthermore, the simultaneous origin of both IPAs from the CeT also complicates procedures such as liver transplantation and oncological resections [4]. For interventional radiologists, this morphology allows for multi-vessel access but increases the risk of non-target embolization during transarterial chemoembolization [4].

## Data Availability

The original contributions presented in the study are included in the article, further inquiries can be directed to the corresponding author.
